# Efficacy and safety of probiotics in preventing chemotherapy-related diarrhea in patients with colorectal cancer: A systematic review and meta-analysis based on 18 randomized trials

**DOI:** 10.1097/MD.0000000000043126

**Published:** 2025-07-04

**Authors:** Meilin Yang, Lu Wang, Chu Luo, Jiarong Shang, Xia Zheng, Jun Qian, Ran Yang

**Affiliations:** a Department of Oncology, Affiliated Hospital of Nanjing University of Chinese Medicine, Nanjing, Jiangsu, China; b Nanjing Jiangning Hospital in Jiangsu Province, Nanjing, Jiangsu, China.

**Keywords:** chemotherapy-related diarrhea, colorectal cancer, meta-analysis, probiotics

## Abstract

**Background:**

Chemotherapy, as one of the main treatments for patients with colorectal cancer (CRC), brings clinical benefits with varying degrees of gastrointestinal reactions. Post-chemotherapy diarrhea is one of the factors affecting the quality of life of cancer patients. In severe cases, it can cause interruption of the chemotherapy process and even be life-threatening. Probiotics’ role in preventing post chemotherapy diarrhea in CRC patients has not been proven.

**Methods:**

This meta-analysis was registered with PROSPERO (CRD42023480526). A comprehensive search using subject terms and keywords was conducted to identify randomized controlled trials. Six databases, CNKI, Wanfang, VIP Journals, PubMed, EMBASE, and Web of Science, were searched from their inception until January 16, 2024. The search terms used included “cancer,” “tumor,” “chemotherapy,” “diarrhea,” “probiotics,” and “placebo.” Clinical studies using probiotics to intervene in post chemotherapy diarrhea in CRC patients were included and data were independently extracted for each study. Meta-analysis and sensitivity analysis were performed using RevMan 5.4 and Stata 14 software.

**Results:**

Eighteen studies involving 1526 patients were included in this analysis. Meta-analysis demonstrated that probiotics significantly reduced the incidence of diarrhea following chemotherapy in the probiotic group compared to the control group (risk ratio, 0.51; 95% confidence interval: [0.40–0.64]; *P* = .029). Meanwhile, the use of probiotics significantly shortened the duration of diarrhea (risk ratio: -2.38, 95% confidence interval: [-2.96 to 1.80]). Furthermore, probiotics have been shown to positively affect other gastrointestinal symptoms. Specifically, probiotics significantly alleviated bloating, nausea/vomiting, loss of appetite, and abdominal pain. However, no significant differences were observed in the effects of probiotics on enteritis, tumor necrosis factor-α, diamine oxidase, and interleukin-6.

**Conclusion:**

The comprehensive analysis of 18 randomized controlled trials provided compelling evidence that the probiotics have significant clinical value in preventing the onset of diarrhea, shortening the duration of diarrhea, and alleviating chemotherapy-induced gastrointestinal symptoms.

## 1. Introduction

Colorectal cancer (CRC) is the third most common cancer globally, as reported by the International Agency for Research on Cancer and the World Health Organization.^[1]^ Patients with early-stage CRC usually present no specific symptoms, and most are in the middle or terminal stages of the disease when they begin to experience symptoms.^[2 ]^ The treatment typically involves chemotherapy and immunotherapy. Chemotherapy-induced diarrhea (CID) is a significant complication of cancer treatment, affecting approximately half of all patients with cancer, especially those undergoing regimens that include irinotecan and fluorouracil.^[3]^ Furthermore, irinotecan-induced delayed diarrhea is particularly concerning, manifesting within 24 hours to 2 weeks of chemotherapy.^[4]^ This condition is characterized by persistent high-frequency diarrhea lasting several days. The mechanisms underlying CID vary among chemotherapeutic drugs; however, they commonly damage the intestinal mucosa, leading to inflammation or ulceration.^[5]^ This results in gut microbiome dysbiosis and abnormal gastrointestinal secretion.^[6]^ Clinical manifestations include abnormal electrolyte retention, dehydration, electrolyte imbalance, malnutrition, psychological stress, and significant disruption of daily life.^[7]^ In severe cases, diarrhea can precipitate circulatory failure, posing a life-threatening risk.^[8]^

Management of CID primarily involves symptomatic care. Dietary intervention and fluid and electrolyte supplementation can address mild cases.^[3]^ More severe instances may require pharmacological intervention with medications such as loperamide or octreotide, which help reduce excessive intestinal motility. However, these medications have potential adverse effects that can negatively influence clinical treatment and are not fully effective in preventing CID.^[9]^ Consequently, there is a need for a straightforward and safe approach to reduce the incidence of CID. Probiotics, beneficial microorganisms that contribute to a balanced human digestive tract microbiome, may enhance immune function and mitigate intestinal inflammation by improving the gut microbiota.^[10]^ Probiotics can reconstitute a disrupted gut microbiome, restore normal gut function, and alleviate gastrointestinal symptoms in patients undergoing chemotherapy. Systematic reviews and meta-analyses have demonstrated that probiotics can decrease the occurrence of diarrhea and intestinal diseases in patients undergoing chemotherapy or radiotherapy.^[11]^ However, the efficacy of probiotics against CID in patients with CRC remains unclear. This study aimed to evaluate the preventive potential of probiotics for CID in patients with CRC.

## 2. Materials and methods

### 2.1. Registration

This study was registered with PROSPERO (registration number CRD42023480526).

### 2.2. Search strategy

Subject terms and keywords were used to search 6 databases. The search terms “cancer,” “tumor,” “diarrhea,” “chemotherapy,” “chemotherapy-related diarrhea,” and “probiotics” were used in CNKI, Wanfang, and VIP Journals databases. “cancer,” “tumor,” “chemotherapy,” “diarrhea,” and “placebo” were used in PubMed, EMBASE, and Web of Science databases. Additionally, references cited in the included studies and related review articles were searched within the timeframe from database inception to January 16, 2024. Two researchers conducted literature searches and screenings, and disagreements were resolved by consulting with a third researcher.

### 2.3. Inclusion and exclusion criteria

#### 2.3.1. Inclusion criteria

The inclusion criteria for this study were based on the Population, Intervention, Comparison, Outcomes, and Study principle: (1) study participants: (a) patients diagnosed with CRC by imaging and pathology; (b) no restriction on age, sex, or nationality; (c) receiving chemotherapy and diagnosed with CID; (d) excluding patients with serious co-morbidities (e.g., cardiovascular disease); (2) intervention: probiotic treatment administered to the experimental group; (3) comparison: the control group was not treated with probiotics, and when diarrhea appeared it was treated according to the National Comprehensive Cancer Network Guidelines guidelines, for example, with medications such as montelukast and loperamide; (4) outcome indicators: primary outcome indicator, incidence of chemotherapy-related diarrhea; secondary outcome indicators, incidence of other gastrointestinal symptoms and adverse events; and (5) study design: efficacy and safety assessment of probiotics based on randomized controlled trials.

#### 2.3.2. Exclusion criteria

The exclusion criteria were as follows: (1) conference abstracts, reviews, case reports, or animal studies; (2) patients with other primary tumors; (3) studies lacking primary outcome indicators; (4) for the same clinical trial, only the most informative and recently published literature was selected to avoid repetition; and (5) insufficient data to meet the inclusion criteria.

### 2.4. Outcome indicators

Primary outcome indicator: incidence of CID, including at ≥ 1 of the following: diarrhea grade, frequency, or duration. Secondary outcome indicators were the incidence of other gastrointestinal symptoms, such as bloating, abdominal pain, loss of appetite, and nausea/vomiting. Adverse events: incidence of conditions such as enteritis and neutropenia.

### 2.5. Literature selection and data extraction

Two researchers conducted the literature selection and data extraction, resolving discrepancies through discussion and full-text review by the team. The extracted data primarily included the following information: first author, publication year, region, age range, sample size, control group, chemotherapy drug, probiotic type, and outcome indicators.

### 2.6. Quality assessment of the literature

Two researchers assessed the quality of the literature using the Review Manager software version 5.4, guided by the Cochrane Handbook for Systematic Reviews of Interventions (version 5.1). The assessment criteria were as follows: (1) method of random allocation; (2) concealment of allocation; (3) implementation of blinding; (4) completeness of the outcome data; (5) selective reporting of results, and (6) other sources of bias. Each study’s bias was categorized as low-, unknown-, or high-risk.

### 2.7. Statistical analysis

Statistical analysis was performed using RevMan 5.4 and Stata 14 software. The risk ratio (RR) and 95% confidence interval (CI) were used as efficacy indicators to analyze the adverse event data. The chi-square test was used to assess the heterogeneity among the included studies. A fixed-effects model was applied for the meta-analysis if *P* > .05 and I^2^ < 50%, indicating no significant heterogeneity. Conversely, if *P* < .05 or I^2^ > 50% suggesting considerable heterogeneity, a random-effects model was chosen, with subsequent subgroup analysis to explore heterogeneity sources.^[12]^ Funnel plots and Egger test were used to detect publication bias.^[13]^ A symmetric distribution of points in the funnel plot around the central axis indicated the absence of publication bias.^[14]^

## 3. Results

### 3.1. Literature search

**Figure 1.**
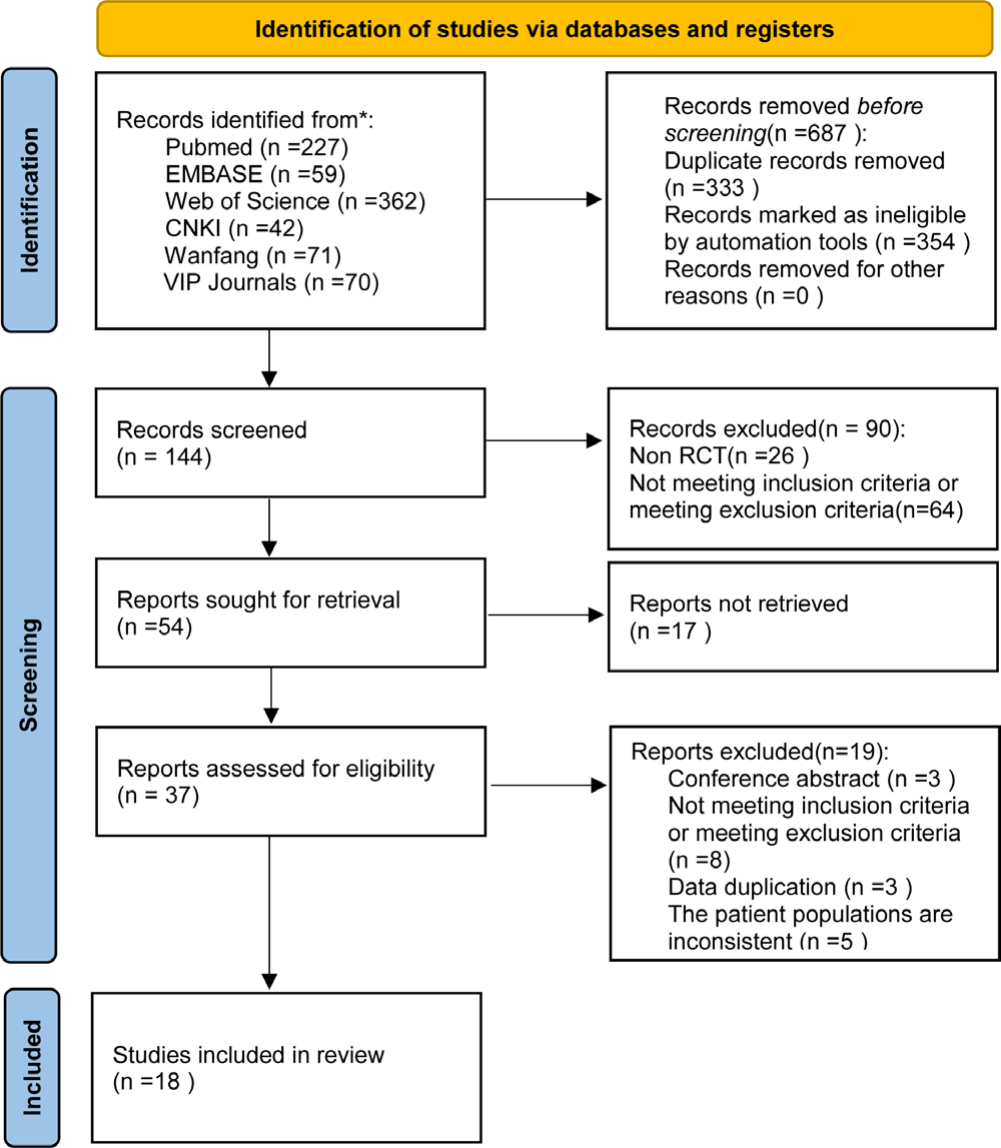
Flowchart of the literature search process for meta-analysis.

After an initial screening of 6 databases, including CNKI, Wanfang, VIP Journals, PubMed, EMBASE, and Web of Science, 831 articles were obtained. Seventy-three studies were selected for further evaluation, and 18 high-quality studies involving 1526 patients were included. The selection process is illustrated in Figure 1.

### 3.2. Basic characteristics of the included studies

This study included 18 randomized controlled trials involving 1526 patients.^[15–32]^ Five studies used a placebo for the control group, whereas 13 used a non-active control group. The probiotic strains varied across trials, with 2 studies using 1 strain, 6 using identical probiotics, and others using 2 or more strains. Table 1 summarizes the characteristics of the included studies. Of the 18 studies, 3 gave irinotecan-based chemotherapeutic agents, 2 used fluorouracil-based chemotherapy regimens, 5 used XELOX regimens, 6 used FOLFOX regimens, 1 used FOLFRI regimens, and 1 study did not describe the use of chemotherapeutic agents.

**Table 1:** Characteristics of the studies included in the meta-analysis.

First author	Year	Country	Age range	Total patient (Trail/Control)	Control	Chemotherapy drug	Probiotic interventions	Outcomes
Michal Mego^[24]^	2015	Slovakia	42–81	23/23	placebo	Based on irinotecan	Bifidobacterium breve HA-129, Bifidobacterium bifidum HA-132 HA, Bifidobacterium longum HA-135, Lactobacillus rhamnosus HA-111, Lactobacillus acidophilus HA-122, *Lactobacillus casei* HA-108, Lactobacillus plantarum HA-119, Streptococcus thermopilus HA-110, Lactobacillus brevis HA-112, Bifidobacterium infantis HA-116	Different grades of diarrhea; Enterocolitis and abdominal distention; Usage of antidiarrheal drugs
Feng Huang^[23]^	2023	China	57–72	50/50	placebo	XELOX regimen	B. infants, L. acidophilus, E. faecalis, and B. cereus	Incidence of diarrhea, gastrointestinal adverse reactions, and gut Bacterial Taxa
P Österlund^[27]^	2007	Finland	31–75	97/51	blank	Mayo regimen/The simplified de Gramont regimen	Lactobacillus rhamnosus GG	Different grades of diarrhea; Incidence of all kinds of Stomatitis, Neutropenia, Neutropenic infection, and Hand-foot syndrome.
Michal Mego^[25]^	2023	Slovakia	29–82	98/100	Placebo	Based on irinotecan	Bifidobacterium,BB-12 and Lactobacillus rhamnosus, LGG	Different grades of diarrhea and enterocolitis; Incidence of abdominal bloating; Diarrhea duration; Usage of antidiarrheal drugs; Number of watery stool, mushy stools
Liyana Zaharuddin^[22]^	2019	Malaysia	58–77	8/6	Placebo	XELOX regimen	Lactobacillus acidophilus, Lactobacillus lactis, Lactobacillus casei subsp, Bifidobacterium longum, Bifidbacterium bifidum and Bifidobacterium infantis	Different grades of diarrhea
Yang Yang^[10]^	2020	China	43–67	40/40	blank	XELOX regimen	Bifidobacterium, Lactobacillus, Enterococcus	Different grades of diarrhea; Incidence of abdominal bloating, neutropenia, Nausea and vomiting; DAO
Lu Helei^[11]^	2019	China	54–72	20/20	Placebo	XELOX regimen	Bifidobacterium longum, Bifidobacterium lactis powder, Lactobacillus acidophilus, Lactobacillus rhamnosus, Lactobacillus paracei, Lactobacillus fermentum	Different grades of diarrhea; Incidence of abdominal bloating, neutropenia, Nausea, and vomiting
Fang Liping^[14]^	2011	China	44–69	18/18	Blank	Based on irinotecan	Bifidobacterium	Different grades of diarrhea, KPS, Flora distribution
Ge Jiansheng^[16]^	2014	China	64–79	35/35	Blank	Not described	Bifidobacterium、lactobacillus、Enterococcus	DAO, TNF-α, total effective rate
Zheng Liang^[12]^	2011	China	41–72	72/72	Blank	Based on fluoropyrimidine	Bifidobacterium, Lactobacillus, Bacillus subtilis, and Bacillus butyrici	Different grades of diarrhea
Wei Huizhang^[13]^	2017	China	40–70	30/30	Blank	FOLFIRI regimen	bifidobacterium	Different grades of diarrhea, Diarrhea duration
Zhan Yuqiang^[17]^	2018	China	40–74	41/42	Blank	mFOLFOX6 regimen	Bifidobacterium longum, Lactobacillus bulgaricus, Streptococcus thermophilus	Incidence of diarrhea; Flora distribution
Zhang Yixuan^[19]^	2020	China	33–58	47/47	Blank	mFOLFOX regimen	Bifidobacterium、Lactobacillus、Enterococcus	Incidence of diarrhea, abdominal bloating, loss of appetite, abdominal pain, neutropenia, Nausea and vomiting; Flora distribution
Liu Hao^[18]^	2019	China	38–72	42/42	Blank	mFOLFOX6 regimen	Bifidobacterium、Lactobacillus、Enterococcus	Incidence of diarrhea, abdominal bloating, loss of appetite, Nausea and vomiting; Flora distribution
Shao Yundi^[26]^	2018	China	55–77	45/45	Blank	mFOLFOX6 regimen	Bifidobacterium, Lactobacillus, Bacillus subtilis, and Bacillus butyrici	Incidence of diarrhea, loss of appetite, Nausea and vomiting; Flora distribution
Zhang Dongmei^[20]^	2013	China	Not described	30/30	Blank	FOLFOX4 regimen	Bacillus lichen、Bifidobacterium、Lactobacillus、Enterococcus	Total effective rate and KPS
Liang Shuwen^[28]^	2014	China	34–71	44/41	Blank	XELOX regimen	Bifidobacterium、Lactobacillus、Enterococcus	Diarrhea duration, Total effective rate, and KPS
Wang Yajun^[21]^	2018	China	54–70	28/28	Blank	FOLFOX4 regimen	Bifidobacterium、Lactobacillus、Enterococcus	Incidence of diarrhea

DAO = diamine oxidase, KPS = KarnofskyPerformance Status, TNF-α = tumor necrosis factor.

### 3.3. Quality assessment

**Figure 2.**
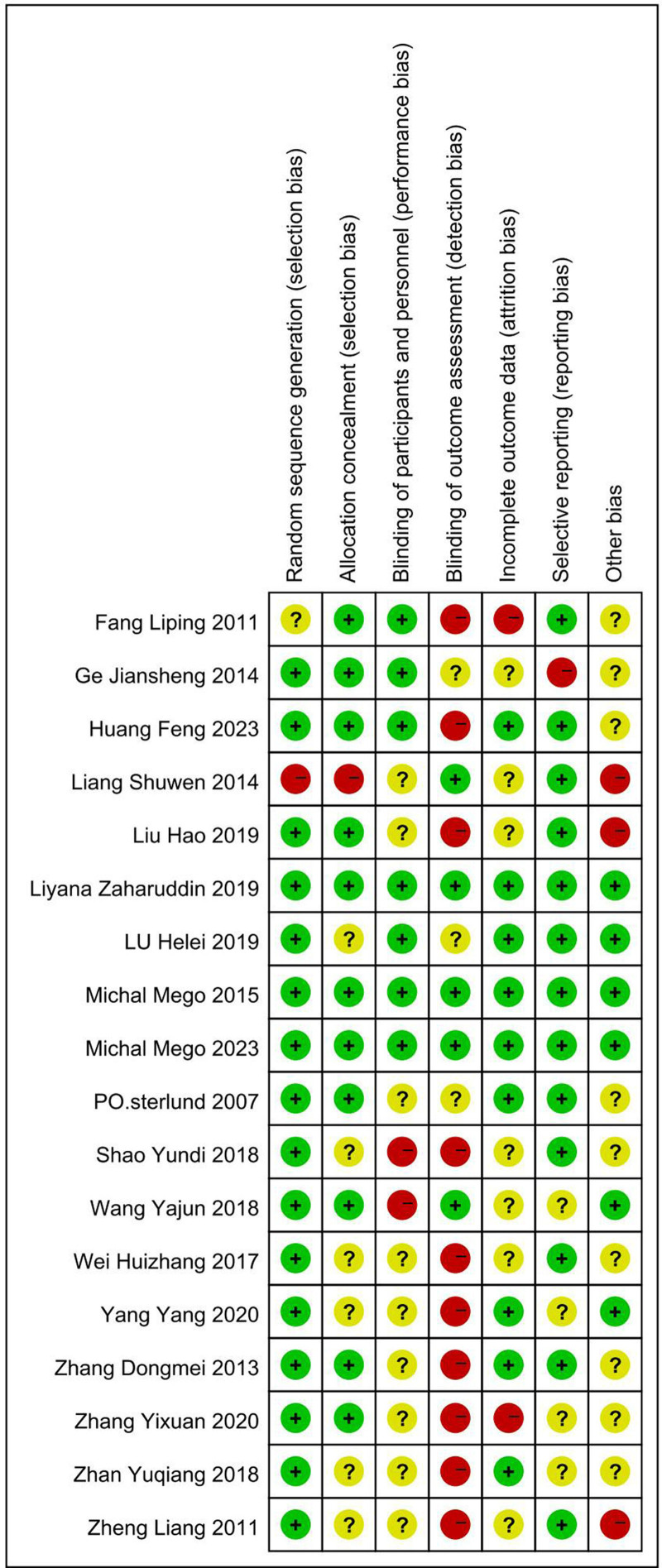
Summary of risk of bias across the included studies.

The bias risk graph (Fig. 2) indicated good overall methodological quality, with blinding as the primary high-risk area. The definitions of adverse events were unclear in many studies, suggesting a potential reporting bias.

### 3.4. Efficacy of probiotics

**Figure 3.**
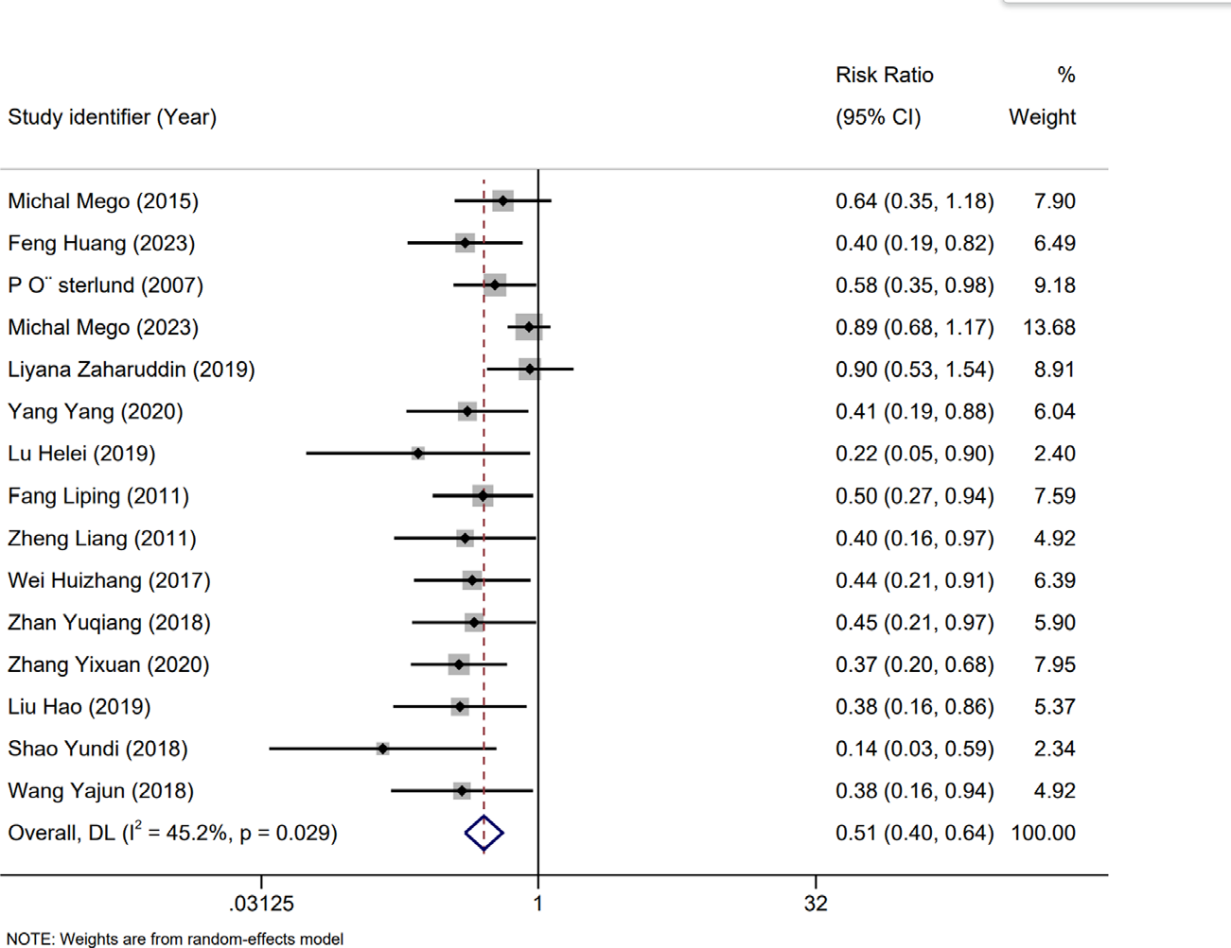
Forest plot for the overall incidence of diarrhea.

**Figure 4.**
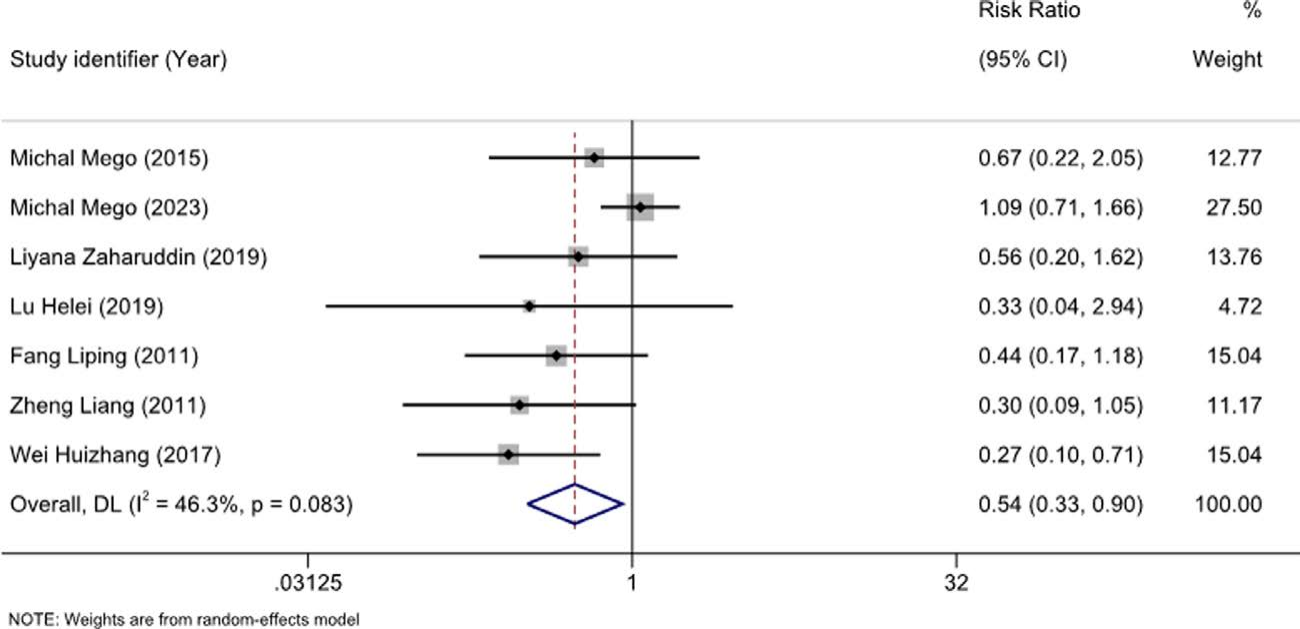
Forest plot for diarrhea with a severity ≥ 2.

**Figure 5.**
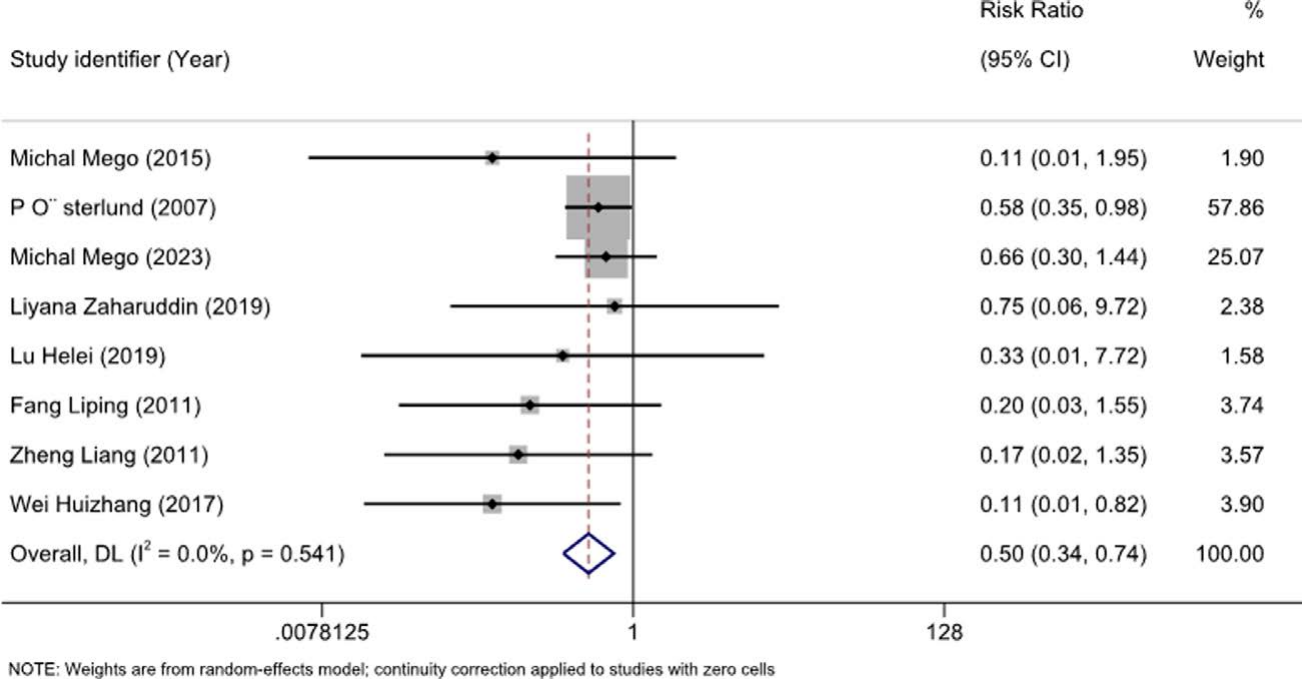
Forest plot for diarrhea with a severity ≥ 3.

We analyzed 15 studies^[15–19,21–23,25–31]^ with 1311 patients to assess the preventive effects of probiotics on CID. Mild heterogeneity was observed (I^2^ = 45.2%; *P* = .029), and a random-effects model was used. Meta-analysis showed a significant reduction in the incidence of diarrhea following chemotherapy (RR: 0.51; 95% CI [0.40–0.64]; *P* = .029) (Fig. 3). Further analyses for ≥ grade 3^[16–19,26,28,29,31]^ (RR: 0.50; 95% CI [0.34–0.74]; *P* = .541) and 2^[16–19,26,28,29]^ diarrhea (RR: 0.54; 95% CI [0.33–0.90]; *P* = .083) showed that probiotics effectively prevented these conditions (Figs. 4 and 5).

### 3.5. Other efficacy and safety of probiotics

In addition to their known benefits, probiotics have been studied for their effects on gastrointestinal symptoms following chemotherapy in patients with CRC. A meta-analysis of 5 studies^[15,23,27–29]^ observed significant alleviation of post-chemotherapy bloating with probiotics (RR: 0.45; 95% CI [0.30–0.69]). This finding is particularly relevant because bloating is a common and distressing symptom in these patients. An analysis of 7 studies^[15,16,21–23,27,30]^ suggested that the probiotic treatment group experienced fewer instances of nausea/vomiting (RR: 0.57; 95% CI [0.40–0.81]), highlighting the potential of probiotics to improve the quality of life during the recovery phase. A meta-analysis of 6 studies^[15,16,21–23,30]^ showed a significant reduction in the incidence of post-chemotherapy loss of appetite with probiotics (RR: 0.43; 95% CI [0.35–0.54]), which could contribute to better nutritional status and overall well-being. Two studies^[16,27]^ indicated that probiotics significantly improved post-chemotherapy abdominal pain (RR: 0.32; 95% CI [0.12–0.84]), a symptom that can be particularly debilitating for patients. However, 2 studies^[28,29]^ found that probiotics had no significant impact on enterocolitis improvement (RR: 0.23; 95% CI [0.04–1.34]), suggesting that the role of probiotics in managing this condition remains unclear. A meta-analysis of 3 studies^[18,28,32]^ demonstrated that probiotics effectively reduced the duration of post-chemotherapy diarrhea (RR: -2.38; 95% CI [-2.96 to -1.80]), underscoring the therapeutic potential of probiotics in managing CID. However, the same analysis showed no significant difference in the effects on diamine oxidase (DAO) (RR: -1.18; 95% CI [-3.02 to 0.66]), tumor necrosis factor-α (RR: -13.81; 95% CI [-32.86 to 5.24]), and interleukin-6 (RR: -2.24; 95% CI [-7.75 to 3.28]), indicating that further research is needed to understand the impact of probiotics on these biomarkers. Additionally, 2 studies^[15,27]^ have indicated that probiotics may increase the incidence of neutropenia (Tables 2a and 2b).

### 3.6. Subgroup analysis

**Figure 6.**
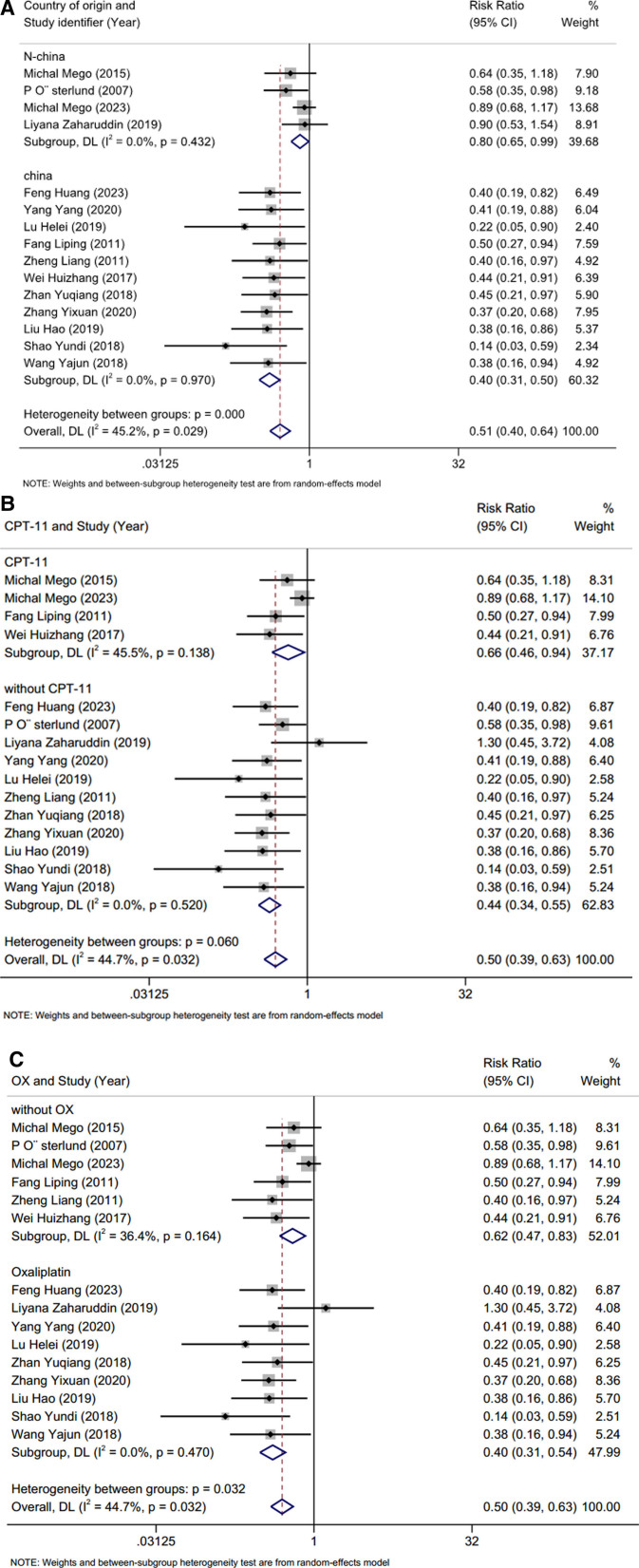
(A–C) Subgroup analysis results for the 2 groups.

The subgroup analysis, stratified by country and using total diarrhea as an indicator, is depicted in (Fig. 6A). These results indicate that this approach reduces heterogeneity. We also used irinotecan and oxaliplatin in the treatment regimens included in the study as indicators of subgroup analysis, and the results are shown in Figure 6B and C. The results of the subgroup analyses showed that country, CPT-11, and oxaliplatin use were all likely sources of high heterogeneity and that probiotics showed similar therapeutic effects in the subgroups. Subgroup analyses showed significant improvement in CID patients treated with probiotics.

### 3.7. Sensitivity analysis

**Figure 7.**
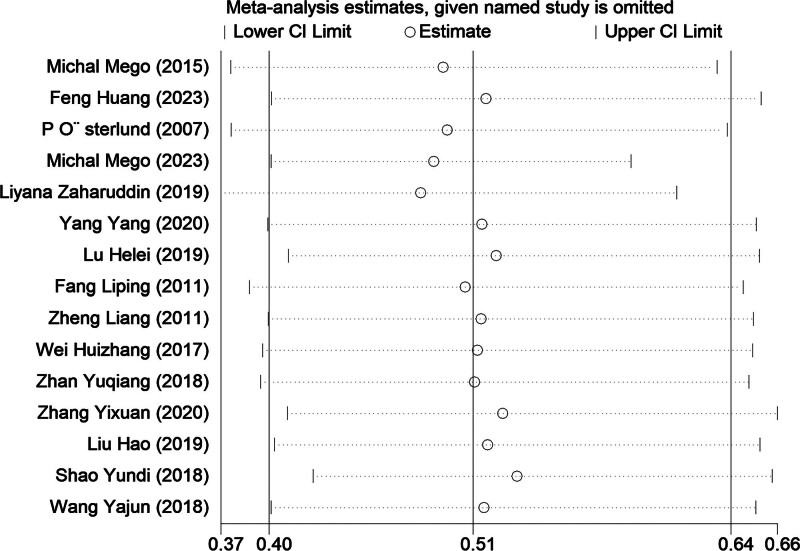
Sensitivity analysis plot for overall diarrhea.

Sensitivity analysis using total diarrhea as the indicator demonstrated that sequentially excluding each study did not alter the 95% CI to cross the line of ineffectiveness. The combined effect size remained within the original 95% CI bounds, suggesting that the study results were significant (Fig. 7).

### 3.8. Publication bias

**Figure 8.**
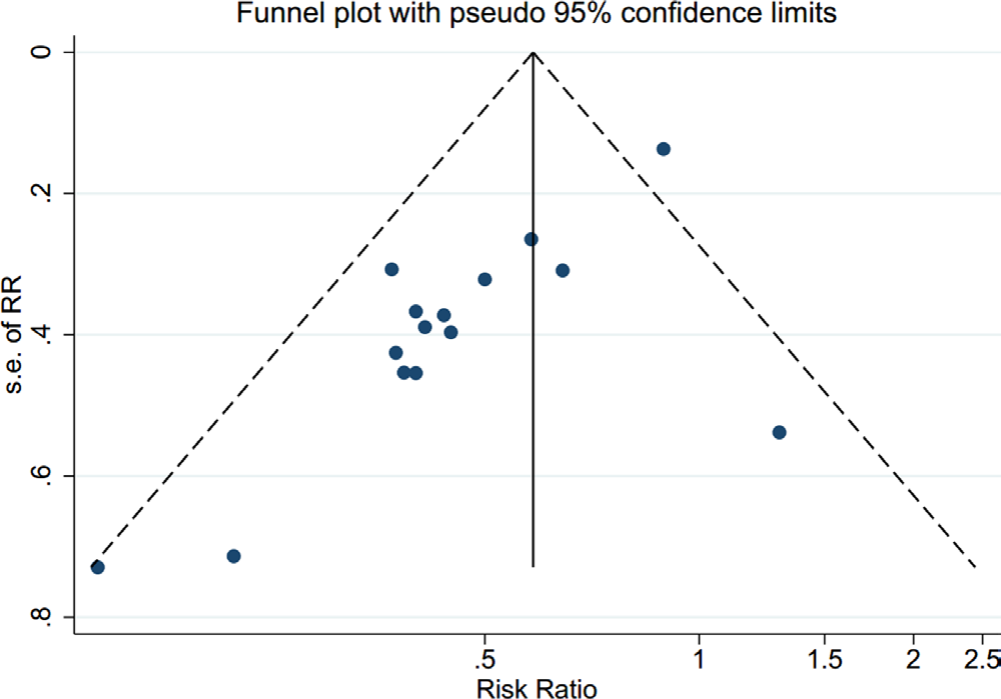
Funnel plot for overall diarrhea.

**Figure 9.**
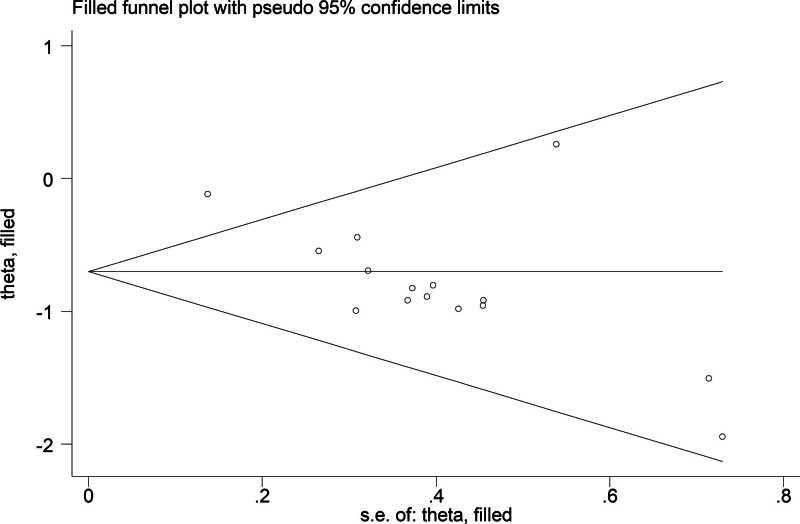
Begg funnel plot after imputation using the trim-and-fill method.

The funnel plot for total diarrhea indicated a publication bias, as evidenced by the Egger test (*P* = .001) (Fig. 8). Re-evaluation using the trim-and-fill method with a fixed-effects model detected no unpublished studies, and the combined effect size was 0.585 (95% CI: 0.498–0.687; *P* < .001). The combined effect size, using a random-effects model, was 0.498 (95% CI: 0.398–0.634; *P* < .001), with the 95% CI remaining significant, confirming the stability and reliability of the study (Fig. 9). No publication bias assessment was conducted for other indicators owing to the few included studies.

## 4. Discussion

In this systematic review and meta-analysis, we evaluated the efficacy and safety of probiotics in preventing CID in patients with CRC. Compared with other meta-analyses, the meta-analysis by Lu et al^[11]^ showed that probiotic treatment could reduce the incidence of total diarrhea and grade ≥ 3 diarrhea, which was consistent with our findings; however, it did not have a significant ameliorative effect on the incidence of grade I and II diarrhea, and there were no reports of adverse events. Feng et al’s^[33]^ meta-analysis showed that the reduction of probiotics to improve chemotherapy-induced side effects occurred only in Asian populations, not in European or American populations, and our study further elucidated the relationship between the occurrence of diarrhea and nationality by expanding the number of studies. A meta-analysis by Lin^[34]^ and others showed that probiotics had an ameliorative effect on diarrhea after radiotherapy or chemotherapy in patients with abdominal and pelvic tumors, especially reducing diarrhea greater than grades 2 and 3; however, this meta-analysis did not include subgroup analyses of cancer types, and its therapeutic role for a single disease type of CRC was not adequately argued; the meta-analysis by Danis et al^[35]^ suggested that oral probiotics failed to be an effective intervention for oncology patients who developed diarrhea after receiving radiotherapy/chemotherapy; a subgroup analysis showed a possible more pronounced effect on patients with CRC who developed CID, but this finding was not further proved. Our study builds on our previous work by documenting in detail the use of chemotherapeutic agents in the study, exploring the therapeutic effects of probiotics in patients with CRC after the occurrence of CID, and reporting possible adverse events. By synthesizing data from 18 randomized controlled trials, we found that probiotics significantly reduced the incidence of CID. Specifically, the probiotic group had a significantly lower incidence of overall diarrhea and grade ≥ 2 and ≥ 3 CID than the control group. This outcome underscores the potential use of probiotics as a preventive strategy against CID. Additionally, probiotics significantly mitigated other chemotherapy-induced gastrointestinal symptoms, including bloating, nausea/vomiting, appetite loss, and abdominal pain. The breadth of these effects highlights the versatile role of probiotics in managing the side effects of chemotherapy. These findings may be related to the potential mechanisms by which probiotics regulate intestinal microecological imbalances and improve gastrointestinal symptoms. To assess the effect of probiotics on intestinal mucosal integrity, we focused on changes in DAO levels, an enzyme closely associated with intestinal mucosal integrity.^[36]^ An analysis of 2 studies, including 150 patients, showed no significant difference in the reduction of serum DAO levels in patients who received chemotherapy, suggesting that the influence of probiotics on intestinal mucosal integrity may not be reflected solely by DAO levels. However, probiotics had a more significant effect in shortening the duration of diarrhea than conventional treatments. These findings have significant implications for improving patient comfort and recovery. No statistically significant improvement in tumor necrosis factor-α and interleukin-6 levels was found. Further research is required to elucidate the effects of probiotics on inflammatory markers.

This meta-analysis also studied adverse reactions related to probiotics, with 2 studies indicating that probiotics may increase the incidence of neutropenia after chemotherapy, raising considerations of patient safety during probiotic use, both of which used 2 chemotherapeutic agents, oxaliplatin and capecitabine. This adverse reaction was not promptly reported in the meta-analysis studies by Wang^[37]^ and Lin et al.^[34]^ It suggests that we should weigh the potential adverse effects of probiotics when considering them as adjuvant therapy, especially the use of oxaliplatin and capecitabine as a chemotherapy regimen should be further considered with caution for the use of probiotics, but this meta-analysis included fewer studies, and this potential risk still needs to be further explored in future studies. Therefore, a cautious approach is recommended until definitive data are available. There are no standardized criteria for reporting adverse reactions across studies. Österlund et al^[31]^ reported that in a probiotic intervention study, 2 of 51 patients in the control group developed neutropenic infections, compared to 9 of 97 patients in the observation group; however, the absence of *Lactobacillus* growth in blood cultures suggests that probiotics did not contribute to these infections, the chemotherapeutic agents used include 5-fluorouracil and calcium folinate. Shuwen et al^[32]^ reported 1 adverse reaction in the observation and control groups; however, the lack of specific descriptions limits the conclusions drawn from these reports, the chemotherapeutic agents used are oxaliplatin and capecitabine.

The 18 studies included in this analysis exhibited moderate heterogeneity with differences in nationality, placebo use, type of bacterial strain, quantity of bacteria, medication duration, patient age, and observed outcomes, which may have contributed to the observed heterogeneity. After subgroup analysis using total diarrhea as an indicator and nationality, CPT-11 and oxaliplatin as indicators, respectively, showed a reduction in heterogeneity, and it was speculated that all 3 indicators might be the source of heterogeneity. Subgroup analysis showed no significant difference in the therapeutic effect of probiotics whether nationality, CPT-11, or oxaliplatin were used as indicators in subgroups, indicating a high degree of consistency in the therapeutic effect of probiotics on CID, which increases the credibility of the results. Sensitivity analysis indicated no qualitative changes in the conclusions when assessing the variations between studies. This consistency reinforces the significance of the findings. Funnel plot analysis revealed significant publication bias in this study, mainly owing to the inclusion of studies with small sample sizes and difficulty in obtaining negative results. reevaluating publication bias using the trim-and-fill method detected zero unpublished articles, and the fixed-effects model combined with the data yielded an effect size of 0.585 (95% CI: 0.498–0.687, *P* < .001), confirming the reliability of the results despite the detected bias.

In summary, this study suggests that probiotics can improve chemotherapy-related diarrhea and alleviate gastrointestinal inflammation and symptoms in patients with CRC. However, this also indicated that probiotics may be a risk factor for reduced neutrophil counts, which are influenced by the type and dosage of chemotherapeutic drugs, necessitating further verification through multiple studies. The potential of probiotics to enhance patient outcomes is clear; however, their use must be balanced with the risk of neutropenia. The limitations of this study are as follows. First, the small sample size of some of the included studies may limit the comprehensive assessment of the effects of probiotics. Further studies with larger cohorts are required to confirm these findings. Second, the types and dosages of chemotherapeutic drugs were not standardized across different studies, which may have affected comparisons of the effects of probiotics. Standardization in future trials could provide more definitive evidence for the benefits of probiotics. Finally, the lack of a standardized recording of adverse reactions makes it difficult to assess the safety of probiotics accurately. The development of uniform reporting standards would significantly enhance the evaluation of probiotic safety. Future studies should conduct large-scale, high-quality, and detailed clinical controlled trials to overcome these limitations and further validate the role of probiotics in preventing CID in patients with CRC. Future studies should focus on the dosage of probiotics, strain selection, treatment duration, and interactions with chemotherapeutic drugs to provide more precise guidance for clinical practice.

**Table 2a:** Other efficacy and safety of probiotics.

Groups/subgroups	Number of studies	Number of participants	Result			
			RR	95% CI	I²	*P* value
Abdominal bloating	5	518	0.45	0.30–0.69	10.0%	.349
Nausea/vomiting	7	571	0.57	0.40–0.81	60.7%	.018
Abdominal pain	2	140	0.32	0.12–0.84	0.0%	.494
Enterocolitis	2	242	0.23	0.04–1.34	0.0%	.906
Neutropenia	2	180	2.81	1.43–5.52	0.0%	.446
Loss of appetite	6	471	0.43	0.35–0.54	3.2%	.396

CI = confidence interval, CID = chemotherapy-induced diarrhea, RR = risk ratio, TNF-α = tumor necrosis factor.

**Table 2b:** Other efficacy and safety of probiotics.

Groups/subgroups	Number of studies	Number of participants	Result			
			MD/SMD	95% CI	I²	*P* value
Duration of diarrhea	3	343	-2.38	-2.96–-1.80	92.7	<.000
TNF-α	2	164	-13.81	-32.86–5.24	84.90%	.01
IL-6	2	146	-2.24	-7.75–3.28	98%	<.0001
DAO	2	150	-1.18^*^	-3.02–0.66	96.00%	.21

CI = confidence interval, CID = chemotherapy-induced diarrhea, DAO = diamine oxidase, IL-6 = interleukin-6, MD = mean difference, SMD = standard mean difference, TNF-α = tumor necrosis factor. * for SMD.

## Acknowledgments

We would like to thank CNKI, Wanfang, VIP Journals, PubMed, EMBASE, and Web of Science databases for their support.

## Author contributions

**Conceptualization:** Meilin Yang, Jun Qian.

**Data curation:** Lu Wang, Chu Luo, Jun Qian.

**Formal analysis:** Lu Wang, Chu Luo.

**Funding acquisition:** Ran Yang.

**Investigation:** Chu Luo, Jiarong Shang, Jun Qian.

**Methodology:** Jiarong Shang, Xia Zheng, Jun Qian.

**Project administration:** Ran Yang.

**Resources:** Ran Yang.

**Software:** Lu Wang, Jiarong Shang, Ran Yang.

**Supervision:** Meilin Yang, Lu Wang, Xia Zheng, Ran Yang.

**Validation:** Meilin Yang, Xia Zheng.

**Visualization:** Jiarong Shang, Xia Zheng.

**Writing – original draft:** Meilin Yang, Lu Wang.

**Writing – review & editing:** Ran Yang, Jun Qian.
